# Nanocomposite Materials Based on TMDCs WS_2_ Modified Poly(l-Lactic Acid)/Poly(Vinylidene Fluoride) Polymer Blends

**DOI:** 10.3390/polym13132179

**Published:** 2021-06-30

**Authors:** Mohammed Naffakh

**Affiliations:** Escuela Técnica Superior de Ingenieros Industriales, Universidad Politécnica de Madrid (ETSII-UPM), José Gutiérrez Abascal 2, 28006 Madrid, Spain; mohammed.naffakh@upm.es

**Keywords:** TMDCs-WS_2_, PLLA, PVDF, nanomaterials, morphology, crystallization, dynamic-mechanical properties

## Abstract

Novel multifunctional biopolymer blend nanocomposites composed of poly(vinylidene fluoride)(PVDF) and tungsten disulfide nanotubes (INT-WS_2_) that are layered transition metal dichalcogenides (TMDCs) were easily prepared by applying an economical, scalable, and versatile melt processing route. Furthermore, their synergistic effect to enhance the properties of poly(L-lactic acid) (PLLA) matrix was investigated. From morphological analysis, it was shown that the incorporation of 1D (INT)-WS_2_ into the immiscible PLLA/PVDF mixtures (weight ratios: 80/20, 60/40, 40/60, and 20/80) led to an improvement in the dispersibility of the PVDF phase, a reduction in its average domain size, and consequently a larger interfacial area. In addition, the nanoparticles INT-WS_2_ can act as effective nucleating agents and reinforcing fillers in PLLA/PVDF blends, and as such, greatly improve their thermal and dynamic-mechanical properties. The improvements are more pronounced in the ternary blend nanocomposites with the lowest PVDF content, likely due to a synergistic effect of both highly crystalline PVDF and 1D-TMDCs nano-additives on the matrix performance. Considering the promising properties of the developed materials, the inexpensive synthetic process, and the extraordinary properties of environmentally friendly and biocompatibe 1D-TMDCs WS_2_, this work may open up opportunities to produce new PLLA/PVDF hybrid nanocomposites that show great potential for biomedical applications.

## 1. Introduction

The biobased, biodegradable aliphatic polyester poly(L-lactic acid) (PLLA) that is derived from natural resources, such as corn and sugar beet, is a highly versatile polymer and a promising alternative to petroleum-derived polymers in many applications as a result of its good biodegradability, renewability, reasonably good mechanical properties, and processability [1,2,3]. Additionally, it is known to be an excellent base polymer for biomedical applications, including drug delivery systems (DDS), sutures, and clips due to the fact that it has good biocompatibility and that its degradation products are benign to the human body. Moreover, unlike other biopolymers like, poly(hydroxyalkanoates) (PHA), poly(ethylene glycol) (PEG), and poly(ε-caprolactone) (PCL), PLLA can be processed using many different methods, such as extrusion, film casting, fiber spinning, and blow molding, due to its higher thermal processability [4]. However, in spite of its many beneficial attributes, historically, its commercial viability has been limited by poor stability during long melt molding and processing cycles, poor production efficiency, and overall high costs. Since the cost and brittleness of PLLA are quite high, it is not economically feasible to use it alone for day-to-day use as a packaging material without blending. Thus, blends of PLLA with several synthetic and biopolymers have been prepared in an effort to enhance the properties of PLLA. PLLA blends with poly(vinylidene fluoride) (PVDF), poly(butylenes succinate adipate) (PBSA), poly(ethylene glycol) (PEG), poly(methyl methacrylate) (PMMA), polypropylene (PP), polyethylene (PE), poly(ethylene oxide) (PEO), and poly(butylenes adipate-co-terephthalate) (PBAT) have been reported to improve the properties, such as toughness, modulus, impact strength, crystallization behavior, and thermal stability, compared to the neat polymer [5,6,7,8,9]. Notwithstanding, successful enhancement in the nucleation and crystallization behavior has been reported for PLLA by immiscible blending with PVDF via epitaxial and interface-assisted nucleation [5]. By using the classical method of fiber extrusion, without any special spinnerets, fibrous structures were obtained consisting of a polylactic acid (PLA) matrix filled with PVDF micro- and nanofibers [10]. This kind of fibers, as a hybrid system, can be successfully used for producing implants and prostheses. PVDF film was widely used in the filtration of protein because of its hydrophobicity being able to reduce surface fouling [11]. In addition, PVDF was also reported as a scaffold for cell culture because the piezoelectric properties can enhance cellular adherence and proliferation [12,13]. Previous studies also found that PLLA can facilitate the α- to β-phase transition of PVDF under eletrospinning and uniaxial stretching [14,15]. Electrospun PLA/PVDF mats exhibited higher cell proliferation for L929 fibroblasts than both PLA and PVDF mats [14]. However, to attain PLA blends with good general properties, typically, some sort of compatibilization strategy is required. For more detailed information on PLA property modification, the recent review by Zheng et al. [16] is recommended, which includes the use of copolymer, reactive polymer, nanoparticle, and low molecular weight chemical addition, as well as interfacial compatibilization, exchange reactions, and dynamic vulcanization. The conventional approach to compatibilizing polymer blends is via the use of copolymers as it is an efficient means to achieve good blend compatibility. However, commercial unavailability of specific copolymers and the fact that they must be synthesized prior to blending is one of its drawbacks [16]. Recent nanotechnology advances have been applied to PLLA-based polymers, resulting in improved chemical, mechanical, and biological properties. Advanced nanocomposite materials were obtained by filling the polymer matrix with both synthetic and natural nanoparticles [17,18,19,20]. The addition of CNTs to polymer composite structures with natural fiber has opened a new era of polymer composites for various structural applications. As polymer matrix reinforcements, different types of CNTs with specific and unique functional groups interact with hydroxyl groups in natural fiber cellulose chains, thus modifying the natural fiber surface [17]. The emerging halloysite-based bionanocomposites are used in applications such as biomedicine, packaging, corrosion protection, and restoration of cultural heritages [19]. In this way, Lisuzzo et al. demonstrated that the chitosan coating of halloysite nanotubes driven by electrostatic interactions can be considered a suitable strategy to obtain drug delivery systems with tunable properties [20]. Of particular interest is the use of layered transition metal dicalcogenide (TMDC) nanostructures, such as tungsten disulfide (WS_2_) and molybdenum disulfide (MoS_2_), which are broadband semiconductors with multidimensional structural anisotropy, 0D (IF), 1D (INT), and 2D [21,22,23,24,25,26]. These environmentally friendly and biocompatible TMDCs nanoparticles also have demonstratable processing, performance, design, and cost advantages over nanoclays, CNTs, etc. when manufacturing advanced biopolymer nanocomposites poly(3-hydroxybutyrate) (PHB), Bio-PNC INT-WS_2_, poly(propylene fumarate) (PPF), poly(ether ether ketone) (PEEK), PLLA, etc. [27,28,29,30]. Furthermore, the cytotoxicity of INT-WS_2_ is comparable to regular environmental particulate matter and much lower than other nanoparticles, like silica or carbon black [31,32]. Moreover, blending nanoparticles with an immiscible mixture (PLLA-blend) expands the possible routes for compatibilization, and unlike polymeric compatibilizers, nanoparticles are not specific to the nature of the immiscible components of the mixture and are easily incorporated by mixing. Further, the addition of nanoparticles can significantly improve the materials’ properties, combining the attributes of the base polymer blend with the characteristics of the nanoparticle [16,33].

The object of the current research is to demonstrate the advantage of using INT-WS_2_ as a suitable nano-reinforcement to improve the performance of promising PLLA/PVDF polymer blends. New melt-processable nanocomposites were prepared via a scalable, versatile, and cheap procedure, without the addition of compatibilizers or modifiers. In particular, the effect of INT-WS_2_ on the morphology, thermal, processability, and mechanical properties of the resulting PLLA/PVDF/INT-WS_2_ nanocomposites is considered.

## 2. Experimental Section

### 2.1. Materials and Processing

Poly(l-lactic acid) (PLLA) and poly(vinylidene fluoride) (PVDF) were purchased from Goodfellow Ltd. (Huntingdon, UK). Multiwall WS_2_ 1D nanotubes (INT-WS_2_) with diameters of 30–150 nm and lengths of 1–20 nm were obtained from NanoMaterials Ltd. (Yavne, Israel) (see Section 3.1). Both the blends and nanocomposites were prepared following the same procedure: each mixture of PVDF and PLLA, with or without INT-WS_2_, was dispersed in a small volume of ethanol (HPLC grade, Sigma-Aldrich Química SL, Madrid, Spain) and homogenized by mechanical stirring and bath ultrasonication for approximately 15 min. Subsequently, the dispersion was partially dried in vacuum at 60 °C under a pressure of about 70 mbar for 24 h. The PLLA/PVDF blends are designated as 80/20, 60/40, 40/60, and 20/80, where the numbers indicate the weight percentages of PLLA and PVDF, respectively. In the case of PLLA/PVDF/INT-WS_2_ nanocomposites, the INT-WS_2_ fraction was 0.5 wt.% of the total composite weight and the ratio of PLLA and PVDF was the same as in the binary blends (80/20-INT(79.6/19.9/0.5), 60/40-INT(59.7/39.8/0.5), 40/60-INT (39.8/59.7/0.5), and 20/80-INT(19.9/79.6/0.5)). In a previous study [30], the crystallization behavior and mechanical properties of PLLA filled with different amounts of INT–WS_2_ (0.1 wt.%, 0.5 wt.%, and 1.0 wt.%) were investigated, and it was found that 0.5 wt.% loading led to the highest property improvements. Therefore, 0.5 wt.% INT–WS_2_ was chosen as an optimum concentration to prepare the ternary PLLA/PVDF/INT–WS_2_ nanocomposites. For the sake of comparison, reference samples of PLLA/INT-WS_2_ (0.5 wt.%) (PLLA-INT) and PVDF/INT-WS_2_ (0.5 wt.%) (PVDF-INT) nanocomposites were also prepared in the same way. The melt-mixing of the resulting dispersions (~6 g) was performed using a micro-extruder (Thermo-Haake Minilab system) operating at 190 °C with a rotor speed of 100 rpm for 10 min. Then, the samples were pressed into films of 0.5 mm thickness in a hot press system using two heating/cooling plates.

### 2.2. Characterization Studies

The morphology of the samples was characterized using an ultra-high field-emission scanning microscopy (FESEM) (SU8000, Hitachi Co., Tokyo, Japan). Cryogenically fractured surfaces from film specimens were coated with a ~5 nm Au/Pd layer to avoid charging during electron irradiation.

Wide-angle x-ray diffraction (WAXS) analysis were carried out using a Bruker D8 Advance diffractometer (Bruker AXS GmbH, Karlsruhe, Germany) and a Ni-filtered CuKα radiation source. Diffractograms were recorded at a 0.2 °/s scan speed and a resolution of 40 points/degree over the 2θ region of 5 to 35 °C. Diffractograms were also recorded at a scanning rate of approximately 7 °C/min to understand the dynamic crystallization and melting behavior of the samples. This was performed by initially holding them at a temperature of 190 °C for 5 min to erase any thermal history, and then cooling from 190 to 30 °C, followed by reheating them over the same temperature range and rate.

Thermogravimetric analysis (TGA) was performed using a TA Instruments Q50 thermobalance (Waters Cromatografía, S.A., Cerdanyola del Vallès, Spain) under nitrogen gas (flow rate = 60 mL/min) at 10 °C/min, over a temperature range of 100–800 °C.

Differential scanning calorimetry (DSC) was performed on a Perkin Elmer DSC7/7700 differential scanning calorimeter (Perkin-Elmer España SL, Madrid, Spain), calibrated with indium (*T*_m_ = 156.6 °C, Δ*H*_m_ = 28.45 kJ/kg) and zinc (*T*_m_ = 419.47 °C, Δ*H*_m_ = 108.37 kJ/kg) under the flow of nitrogen gas (25 mL/min). The samples were first heated to 220 °C and held at the same temperature for 5 min to erase the thermal history. Then, the crystallization of the samples was carried out by cooling from 220 to 40 °C, followed by heating cycles at 10 °C/min over the interval of temperatures between 40 and 220 °C. The crystallization/melting enthalpy of PLLA in the blend nanocomposites was determined by considering the weight fraction of PLLA in the nanocomposites.

Dynamic mechanical analysis (DMA) was performed on rectangular shaped samples using a Mettler DMA 861 device (Mettler-Toledo, Greifensee, Switzerland), at three frequencies of 0.1, 1, and 10 Hz in the tensile mode. An 8 N oscillating dynamic force using an amplitude of 17 μm at fixed frequency was adopted. The relaxation spectra were recorded over the temperature range −100 °C to 150 °C, at a heating rate of 3 °C/min.

## 3. Results

### 3.1. Morphology

Tailoring phase morphology of immiscible blends through the addition of nanoparticles is a universally accepted strategy of forming compatible nanocomposites polymer blends, and typically results in improved upon physical properties. The reason for this is that the added nanoparticles typically locate at the interface of the polymer domains and act as interfacial modifiers, strengthening the interfacial adhesion. Nanoparticles can induce the formation of fine dispersed phase particles from coalescence during melt processing, thus stabilizing the fine morphology and therefore maintaining the properties of the blend.

**Figure 1:**
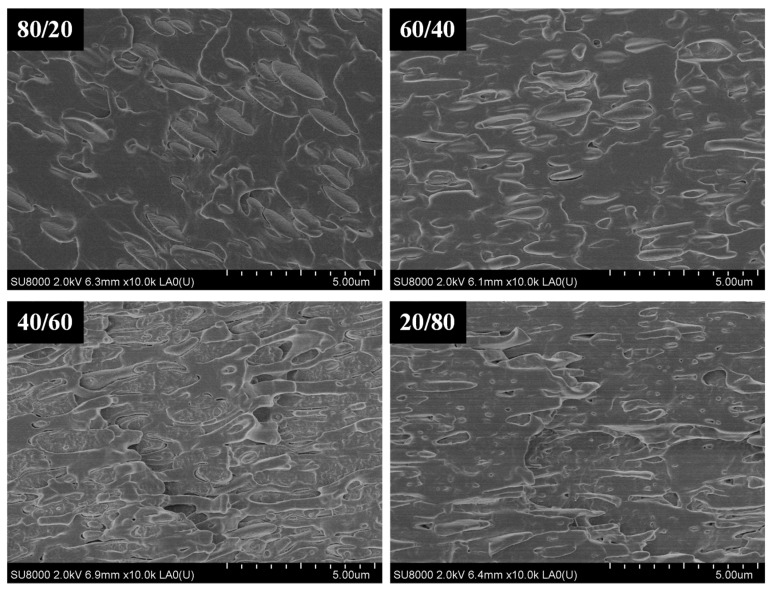
High-resolution SEM image for PLLA/INT-WS_2_, PVDF/INT-WS_2_, binary PLLA/PVDF, and ternary PLLA/PVDF/INT-WS_2_ hybrid nanocomposites.

**Figure 2:**
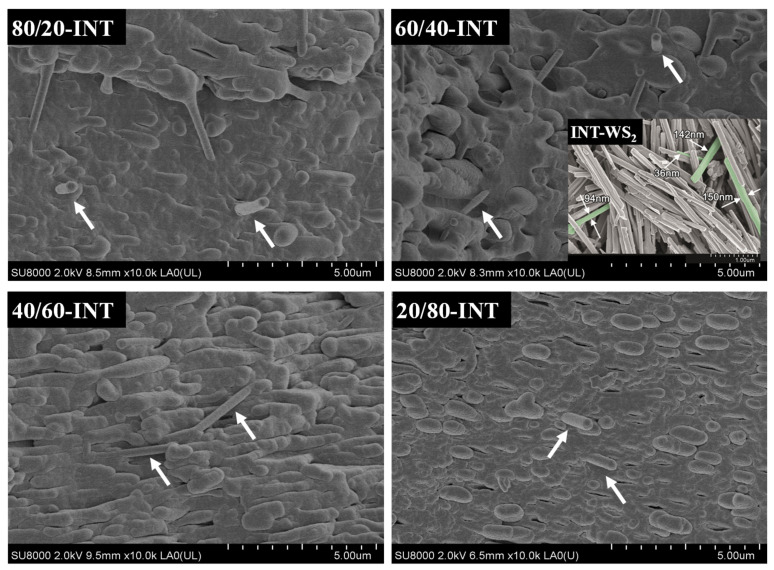
High-resolution SEM image for ternary PLLA/PVDF/INT-WS_2_ hybrid nanocomposites; the inset shows the nanotube dimensions (thicknesses) of as received INT-WS_2_.

The cryogenically fractured surface morphologies using SEM of the PLLA/PVDF blends can be seen in Figure 1. For the 80/20 PLLA/PVDF mixture, a distinct two-phase morphology was noted, with the PVDF phase dispersed evenly within the PLLA matrix. Furthermore, the mean diameter of the domains augmented with increasing PVDF content. When the PLLA content < 60 wt.%, the phase morphology was reversed and the PLLA phase became dispersed in the PVDF. For the ternary hybrid PLLA/PVDF/INT nanocomposites and individual PLLA/INT and PVDF/INT nanocomposites (data are not shown), it was found that the INTs were uniformly dispersed at the nanoscale without evidence of aggregates or agglomerates (see arrows pointing to individual INT-WS_2_ tubes in the images), verifying the effectiveness of the melt extrusion conditions (Figure 2). The INT-WS_2_ nanoparticles also improved the compatibility of the two phases, as demonstrated by an important reduction in the size of the dispersed PVDF domain. This can be attributed to the formed morphological structure in which INT-WS_2_ was mainly dispersed in the PLLA matrix and at the PLLA/PVDF interface.

To achieve similar morphology, other authors employed more elaborate methodologies. For example, carboxyl functionalized multi-walled carbon nanotubes (MWCNTs) were used by Wu et al. to improve the compatibility of immiscible PLA/PCL mixtures [34]. In the case of PLA and PBS, Chen et al. employed double functionalized organoclay (TFC) to increase phase compatibility [35]. They also found that the concentration of TFC had a significant effect on the blend morphology, though when the TFC content < 0.5 wt.%, the PBS domain did not change in size and it was almost exclusively found within the PLA regions. Another nanoparticle that has been used to make immiscible PLA mixtures compatible is silica, as reported by Odent et al. in a mixture of PLA with a gummy copolyester based on 3-caprolactone, P[CL-*co*-LA] [36]. It was found that P[CL-*co*-LA] with spherical nodules dispersed regularly in the PLA matrix in a blank mixture containing 10% by weight of P[CL-*co*-LA], while surface treated (5 wt.% hexamethyldisilazane) spherical nodules disappeared. On the other hand, the compatibility of hydroxyl functionalized and poly(3-caprolactone)-*b*-poly(l-lactide) diblock copolymer grafted polyhedral oligomeric silsesquioxane (POSS-OH and POSS-PCL-*b*-PLLA, respectively) on PLA/PCL blends were analyzed by Monticelli et al. [37]. In both cases, the adhesion between PLA and PCL increased, and with POSS-PCL-*b*-PLLA, a nearly homogeneous microstructure was formed.

### 3.2. Thermal Stability

**Figure 3:**
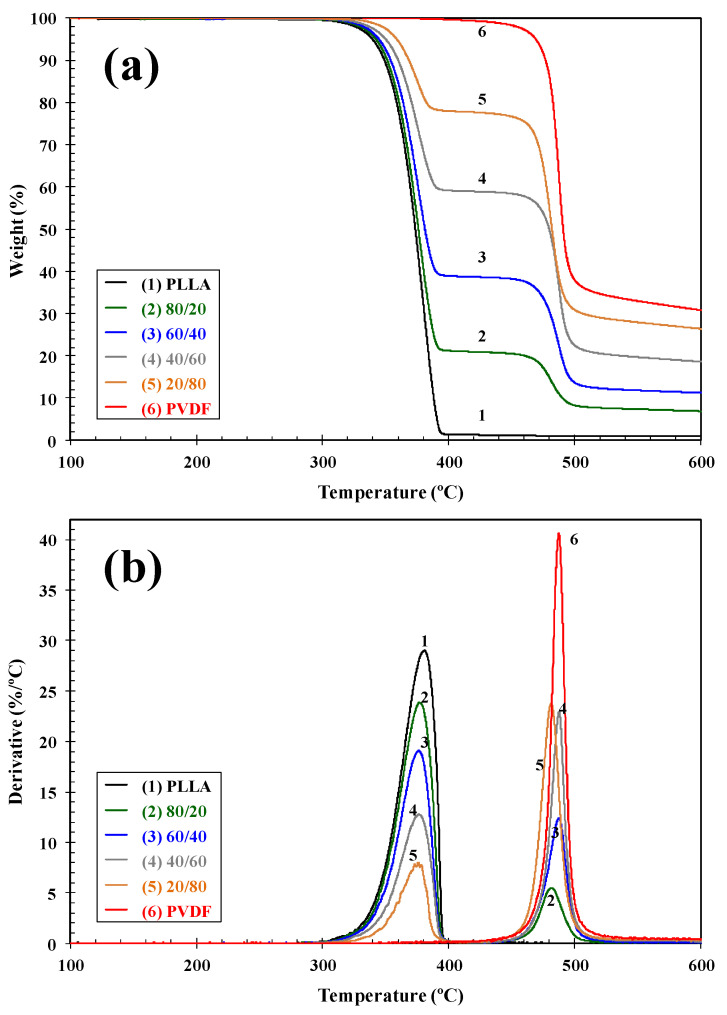
(**a**) Thermogravimetric (TGA) and (**b**) derivative thermogravimetric (DTG) curves of PLLA/PVDF blends.

The thermal stability conditions the processing limits of this type of system, and as such, the study of the effect of blend composition on the degradation temperatures is of utmost importance. Figure 3 shows the variation of the integral and differential thermogravimetric curves, TGA and DTG, of PLLA/PVDF blends as a function of the blend composition. The initial weight loss of PLLA started at around 326 °C, and in the case of PVDF, at a temperature about 100 °C higher. The differential curves show a single peak for each polymer which implies that the degradation processes occurred in a single step. The values of the characteristic degradation temperatures *T*_i_ (temperature for 2% weight loss), *T*_10_ (temperature for 10% weight loss), *T*_max_ (temperature corresponding to the maximum rate of weight loss), and *R*_max_ (rate of maximum decomposition) are summarized in Table 1.

**Figure 4:**
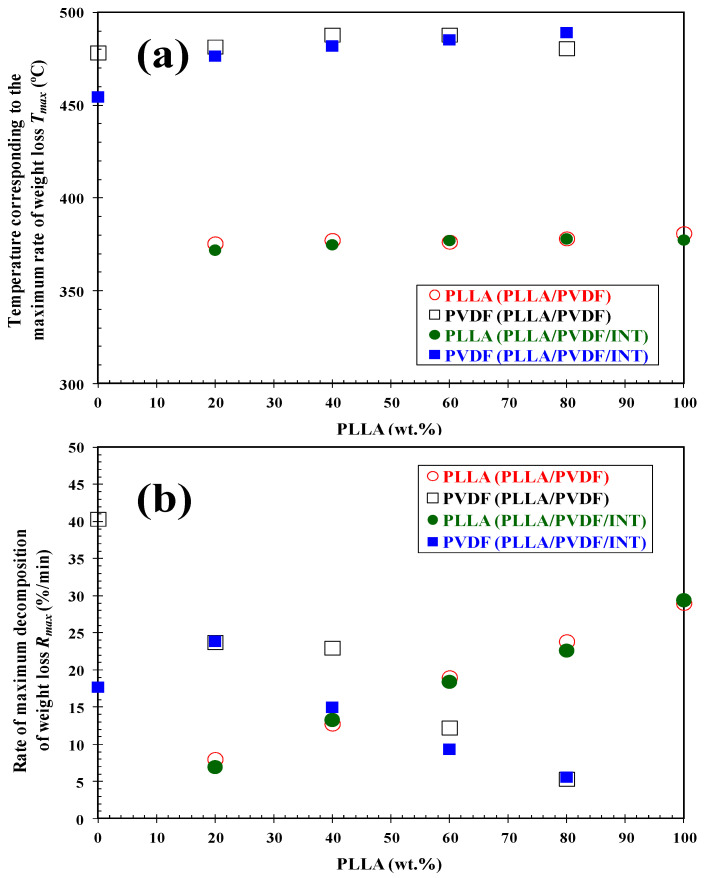
(**a**) Variation of the temperature and (**b**) rate of maximum decomposition (*T*_max_/*R*_max_) of PLLA and PVDF in the binary PLLA/PVDF and ternary PLLA/PVDF/INT-WS2 hybrid nanocomposites with composition.

**Figure 5:**
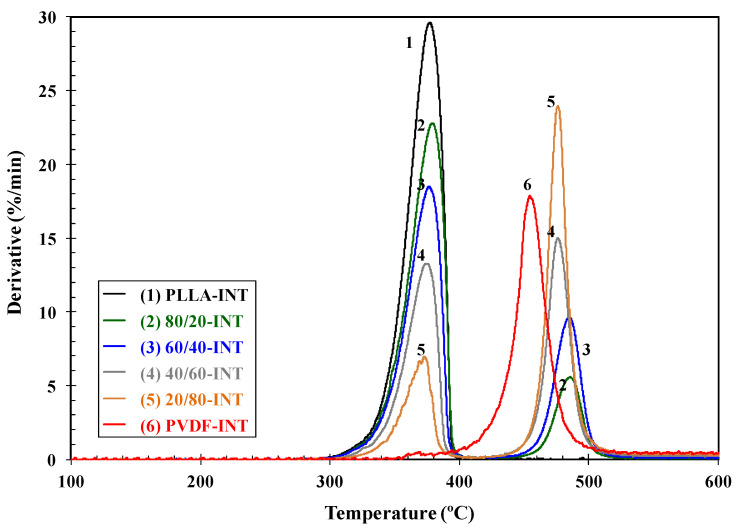
DTG curves of PLLA/PVDF/INT-WS_2_ nanocomposites.

It is clear that the presence of PVDF influenced the degradation behavior of PLLA and vice versa. The addition of PVDF to PLLA hardly increased the thermal stability of the PLLA biopolymer (*T*_i_ and *T*_10_), and the thermal degradation of both components took place via different mechanisms, since two well-defined decomposition stages can be observed. Although the change in *T*_max_ values was small (Figure 4a), the change in the corresponding values of maximum rate of decomposition (*R*_max_) is representative of the variation in the composition of the blends, Figure 4b. In particular, the *R*_max_ of PLLA/PVDF blends clearly fell dramatically with respect to the values observed of neat PLLA and PVDF. Analogously, the addition of 0.5 wt.% INT-WS_2_ to the PLLA/PVDF blends slightly affected the thermal stability (Table 1), without apparent perturbation of the mechanisms of degradation of the blend components, as can be deduced from the presence of the degradation maxima corresponding to PVDF and PLLA (Figure 5).

### 3.3. Crystallization Behavior

DSC was used to study the crystallization behavior of the biopolymers and it was found that PVDF, despite having a similar melting point to PLLA, crystallizes at a faster rate and has a higher crystallization temperature [5]. It can be understood therefore that the PVDF will separate from the melt and crystallize first during cooling. Any modification to the PLLA/PVDF domain interface would affect this process of phase separation, crystallization rate, and hence, crystal morphology.

**Figure 6:**
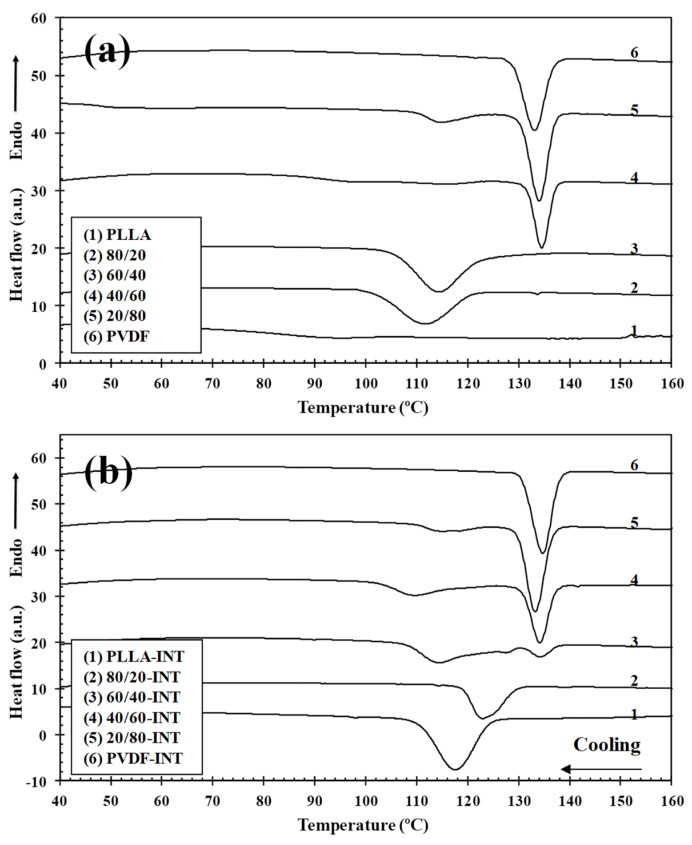
DSC thermograms of the non-isothermal crystallization of (**a**) binary PLLA/PVDF and (**b**) ternary PLLA/PVDF/INT-WS_2_ hybrid nanocomposites obtained during cooling from the melt to room temperature at 10 °C/min.

**Figure 7:**
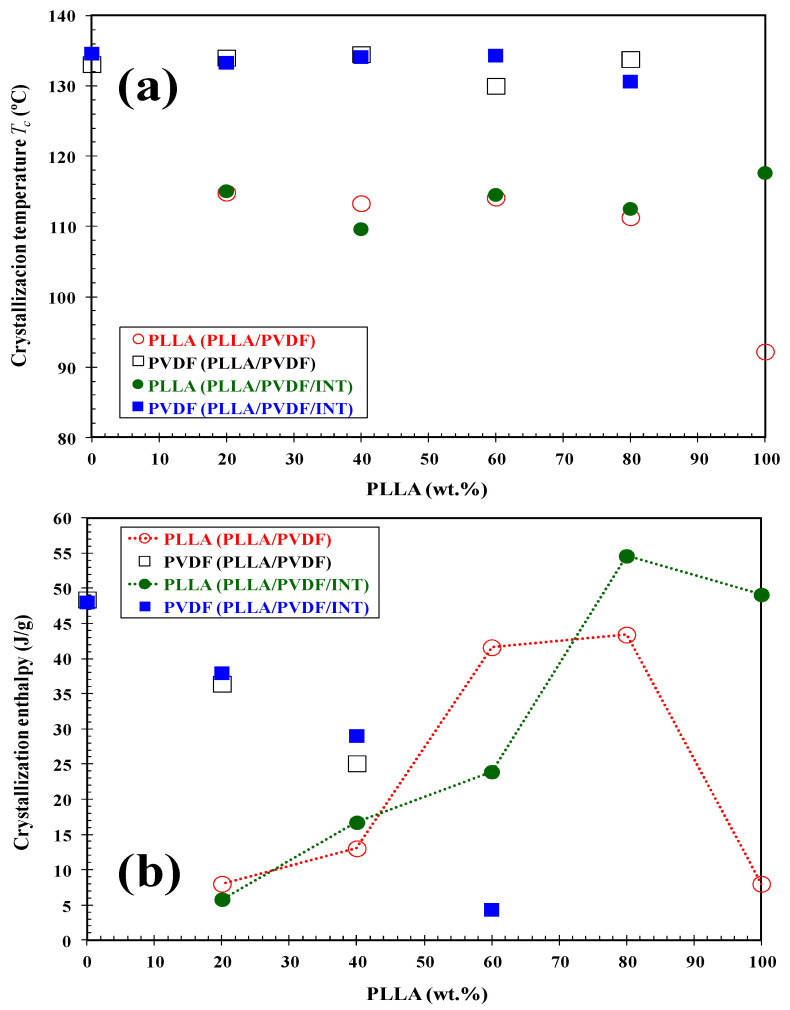
(**a**) Variation of the crystallization temperature (*T*_c_) and (**b**) crystallization enthalpy (∆*H*_c_) of PLLA and PVDF in the binary PLLA/PVDF and ternary PLLA/PVDF/INT-WS_2_ hybrid nanocomposites with composition.

Dynamic DSC cooling scans at a rate of 10 °C/min of the prepared PLLA/PVDF blends (Figure 6a) show that as the ratio of PVDF increased, the PLLA crystallization exotherms shifted to higher temperatures and had a higher enthalpy. It should be noted that the crystallization exotherm of pure PLLA with a crystallization enthalpy of only 8.0 J/g can be difficult to detect. For comparison, the samples containing only 0.5 wt.% INT-WS_2_ are shown in Figure 6b, where it is seen that the crystallization exotherms shifted toward higher temperatures and the enthalpy increased, and in the case of neat PLLA, up to 41.6 J/g. To be able to compare this change in *T*_c_ vs. PLLA concentration for the PLLA/PVDF blends and those containing INT-WS_2_ nanoparticles, Figure 7a is presented. Regarding the blends, a strong increase in the crystallization temperature of PLLA was found upon increasing PVDF content, from 92.2 °C for neat PLLA to 133.1 °C for raw PVDF. A noticeable increase up to 111.3 °C was already found with the incorporation of only 20 wt.% PVDF, but no significant change in *T*_c_ was observed with a further increase of PVDF content. The *T*_c_ of PLLA increased dramatically after blending with PVDF, indicating that PVDF accelerated the melt-crystallization of PLLA. In the same way, the presence of INT-WS_2_ caused an increase in the crystallization temperature, both in the neat polymers and in the blends, the rise being higher than 25 °C for the neat PLLA and about 2 °C for the neat PVDF (see Table 2). With regard to the nanocomposites, this aspect was also observed, though it seems to be less dependent on the concentration of the polymers. As such, it would suggest that the nanofillers provoked nucleation in both polymeric components, with the effect being more pronounced for PLLA. In contrast, 2D-WS_2_ nanosheets have been shown to slow down the crystallization rate of PLLA [25]. Such differences suggest that the nanoparticle shape plays a fundamental role in PLLA crystallization. This discrepancy is likely related to several factors, including the nanofiller geometry, its surface energy, roughness, and crystalline structure as well as on the filler ability to form the critical nucleus [17,22,23]. It should be noticed that, in the case of the 60/40-INT nanocomposite, a double crystallization exotherm was found (Figure 6b), with *T*_c_ values of 114.1 °C and 134.5 °C, which is likely related to the presence of two distinct macrophases, one containing the majority of the 0.5 wt.% INT-WS_2_ and the other encompassing very little. This tendency was also observed for the 40/60-INT and 20/80-INT nanocomposite samples.

**Figure 8:**
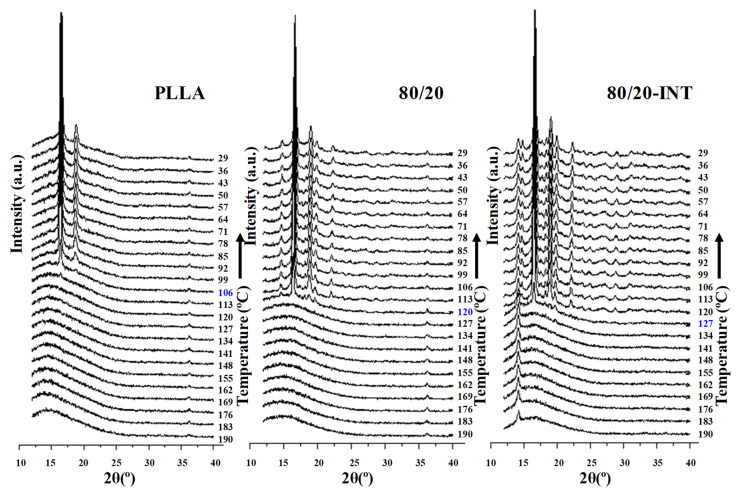
WAXS diffractograms of the dynamic crystallization of PLLA, PLLA/PVDF (80/20), and PLLA/PVDF/INT-WS_2_ (80/20-INT) nanocomposites.

The previously mentioned nucleation effect that led to the increase in crystallization temperature is highly important, particularly when evaluating the crystallization enthalpy tendency. This can be seen in Figure 7b where the variation of the crystallization enthalpy (∆*H*_c_) versus the PLLA wt.% of the blends and the nanocomposites is presented, with the parameters of crystallization detailed in Table 2. For the materials without nanoparticle addition, the value of Δ*H*_c_ reduced from 48.4 J/g for PVDF, that is 47% crystalline (Δ*H*_100 PVDF_ = 103 J/g for perfect crystals [38]) to 8.0 J/g for PLLA, with 8.6% crystallinity (Δ*H*_100 PLLA_ = 93 J/g [39]). However, as can be seen, the increased presence of PVDF provoked a significant increment in the Δ*H*_c_ of PLLA due to it helping to speed up PLLA’s overall crystallization rate. This is reflected in both an increase the temperature of crystallization from melt and enthalpy of crystallization and cold-crystallization suppression (see next section). Using a similar PLLA/PVDF blend, the crystal nucleation was also investigated by Pan et al., where they found that transcrystallization of one polymer type can occur on the crystalline surface of the other polymer [5]. Based on this, it is suggested that the PVDF presence in the immiscible blends promoted PLLA crystallization via two routes, interface-assisted and heterogeneous epitaxial nucleation. The interface between the two phases reduce the surface free energy, facilitating crystal nuclei to form via heterogeneous nucleation. In addition to PVDF crystallization, phase separation can bring about the molecular ordering, alignment, and/or orientation of PLLA chains at the PLLA/PVDF domain interface via interdiffusion, further aiding crystal embryo development [5]. Regarding the samples containing INT-WS_2_, ∆*H*_c_ was observed to increase dramatically as a result of the nucleating effect of the nanoparticles, reaching 54.6 J/g (58.7% crystallinity) for 80/20-INT, the dual-additive system was more important for nucleation than of PVDF alone. Figure 8 illustrates the WAXS profiles of neat PLLA, 80/20, and 80/20-INT recorded during cooling from the melt to room temperature. The characteristic peaks of α-form of PLLA in WAXS patterns (see the following part) appeared as soon as the material attained an appreciable degree of crystallinity. The appearance of these peaks relates well to the crystallization temperature calculated from DSC curves. These results also indicate that the presence of INT-WS_2_ accelerated the crystallization rate of PLLA in the PLLA/PVDF-INT blend nanocomposites. This led to the appearance of the characteristic of the crystalline diffraction of PLLA at higher temperature. Nucleating effects due to the presence of nanofillers have previously been reported for PLLA filled with inorganic nanotubes, nano-calcium carbonate, nano-zinc citrate, graphene oxide and fullerenes (C_60_), nanoclay, and carbon nanotubes [30]. In particular, it was shown that INT-WS_2_ exhibited much more prominent nucleation activity on the crystallization of PLLA than other specific nucleating agents or nano-sized fillers.

### 3.4. Melting Behavior

The melting of semicrystalline thermoplastics is a very complex process significantly influenced by the crystallization conditions. In some circumstances, two peculiarities are observed in the DSC heating scans of semi-crystalline PLLA [40,41]. One is the emergence of a small exothermic peak just before the melting peak and the other is the occurrence of a double melting peak, which has usually been interpreted in terms of a pre-exiting morphology and/or reorganization [30,40]. These two phenomena can be well explained by taking into consideration the crystallization conditions in parallel with the α and α’ crystal formation requirements [41]. When PLLA is crystallized at temperatures corresponding to α crystal formation, the small exotherm appearing just before the single melting peak is due to the transformation of disordered α’ crystals to the ordered α-form. On the other hand, a double melting behavior appears when the crystallization temperature is situated in the region of simultaneous α´ and α type formation. For high crystallization temperatures, only α crystals are produced leading to a single melting peak.

**Figure 9:**
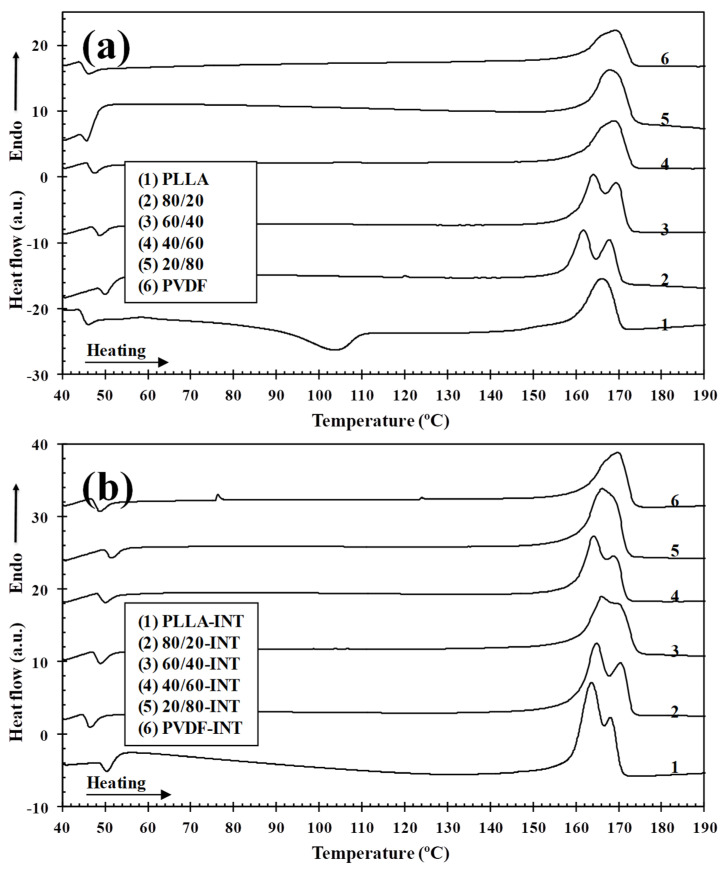
DSC thermograms of melting of (**a**) binary PLLA/PVDF and (**b**) ternary PLLA/PVDF/INT-WS_2_ hybrid nanocomposites obtained during heating at 10 °C/min after cooling from the melt to room temperature at 10 °C/min.

**Figure 10:**
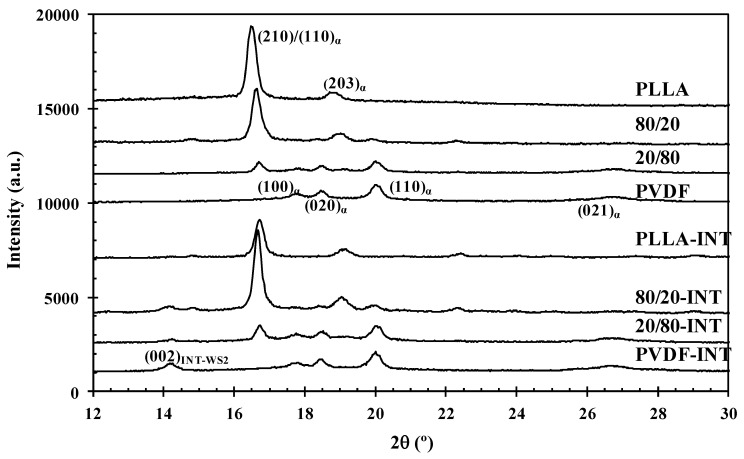
WAXS diffractograms of PLLA, PVDF, binary PLLA/PVDF, and ternary PLLA/PVDF/INT-WS_2_ hybrid nanocomposites obtained at room temperature after dynamic crystallization from the melt.

Figure 9 compares the heating thermograms after cooling from the melt of binary (PLLA/PVDF) and ternary (PLLA/PVDF/INT) hybrid nanocomposites with those of neat PLLA and PVDF. Because the melting peaks of PLLA and PVDF converge in the temperature range of 155−175 °C, the effects of blending on the melting behavior of PLLA cannot be easily identified. It is seen that PLLA presented a maximum endotherm at 166.1 °C of melt after the described exothermic cold-crystallization process. From Figure 10 of the WAXS diffractograms, it can be observed the PLLA sample did not show polymorphism when crystallized under the same conditions as those used for the DSC, with only the main (200)/(110) diffraction peak at 16.7° clearly visible, relating to the PLLA α-form [5,30]. This was similar for the high PLLA content blends, that also principally exhibited the PLLA α-phase characteristic diffraction. In the case of PVDF and high content PVDF blends, diffraction planes of (100), (020), (110), and (021) relating to 2θ = 17.7°, 18.5°, and 20.0° diffraction peaks, respectively were seen, and are typical of the PVDF α-phase [42,43]. In the case when both polymers or polymer blends with nanofiller (INT-WS_2_ [30]) were present, all representative diffraction peaks were observed. From Figure 9b, it can be seen that INT-WS_2_ addition to the mixed PLLA/PVDF blends significantly affected the melting behavior due to the suppression of the cold-crystallization processes as a result of heterogeneous nucleation and the consequences of this as noted earlier [30]. It is also important to note that the Δ*H*_m_ values of the binary (PLLA/PVDF) and ternary (PLLA/PVDF/INT) hybrid nanocomposites were higher than those of neat PLLA and PVDF, which was more distinct for the PVDF-rich blends. This is ascribed to the positive effects of both PVDF and INT-WS_2_ on PLLA crystallization.

### 3.5. Dynamic Mechanical Analysis

**Figure 11:**
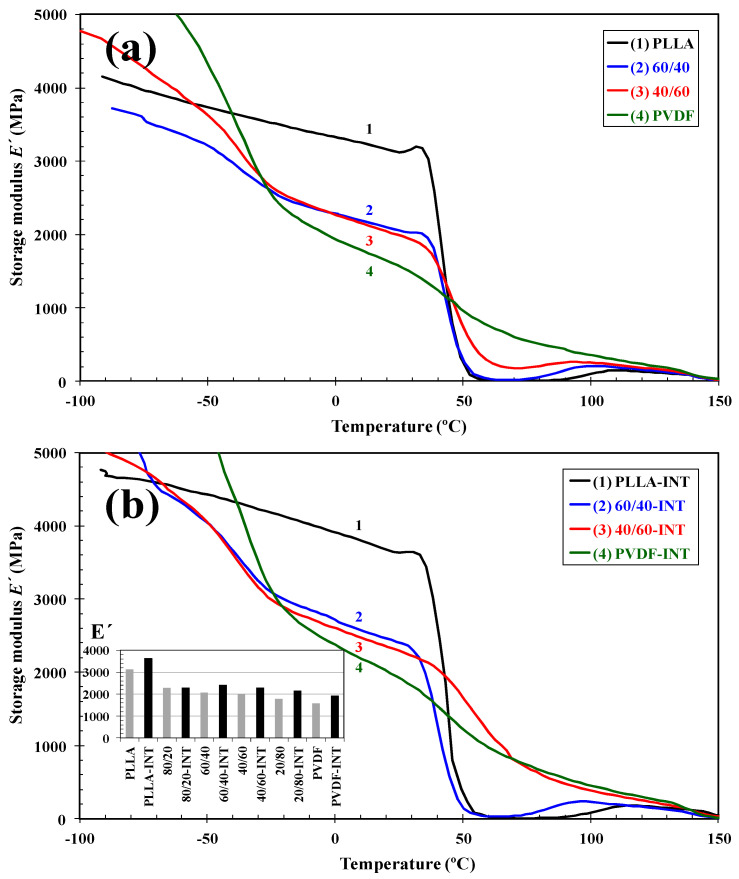
Evolution of the storage modulus (*E′*) as a function of temperature for indicated (**a**) binary PLLA/PVDF and (**b**) ternary PLLA/PVDF/INT-WS_2_ hybrid nanocomposites obtained in the tensile mode at 1 Hz; inset is the room temperature values of storage modulus (*E′*) obtained for all binary and ternary hybrid nanocomposites.

**Figure 12:**
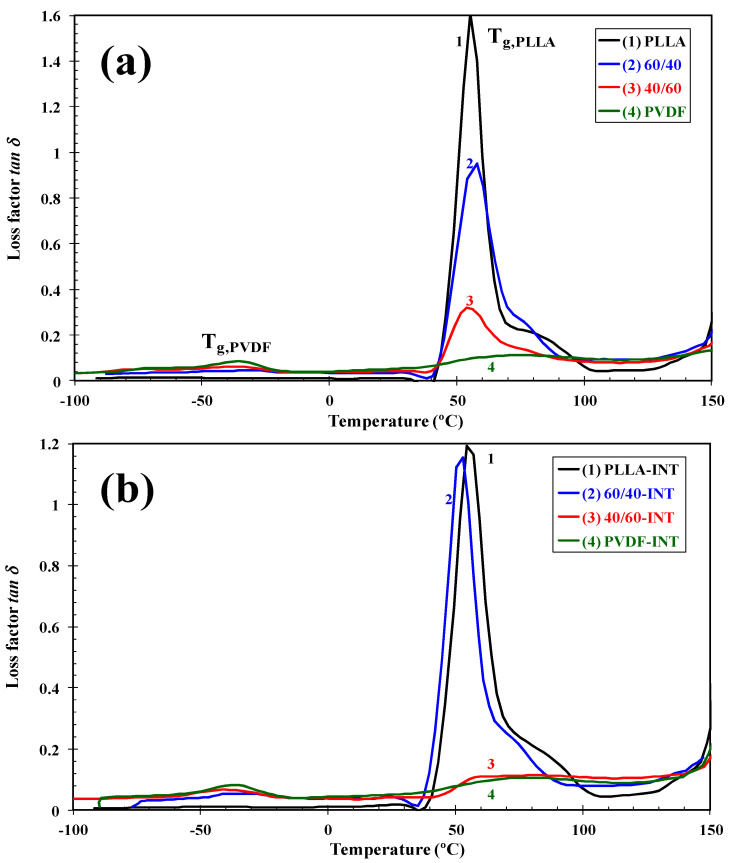
Evolution of the loss factor (*tan δ*) as a function of temperature for indicated (**a**) binary PLLA/PVDF and (**b**) ternary PLLA/PVDF/INT-WS_2_ hybrid nanocomposites obtained in the tensile mode at 1 Hz.

DMA is a technique widely used to characterize a material’s physical properties, such as its glass transition, but is also sensitive to other relaxation processes, making it particularly relevant to assess the impact the addition of the nanofiller has on these events. For example, the storage modulus (*E′*) and loss tangent (*tan δ*) curves for pure PLLA, PVDF, and their binary and ternary nanocomposite blends, prepared by quenching from the melt state, are shown as a function of temperature (see Figure 11 and Figure 12, respectively). It can be seen that between −70 to −20 and 40 to 70 °C, the *E′* of the blends decreased sharply as they passed through the glass transition regions of PVDF and PLLA, respectively. After this, in the temperature ranging from 80 to 140 °C, the *E′* of the blends rose slightly due to the PLLA component cold crystallizing (Figure 11a). Below the *T*_g_ of PVDF as its composition increased in the blend, *E′* also improved according to the rule of mixtures. However, between the *T*_g_ of the two polymers, *E′* was seen to decrease with increasing PVDF content, as at this point the PVDF had transitioned from a leathery to rubbery state. The *T*_g_ and *E′* (at 25 °C) values for all the samples are presented in Table 3, where it is seen that with increasing PLLA content, *E′* increased due to it having a higher modulus than PVDF at this temperature. The addition of the INT-WS_2_ nanofiller to the samples resulted in them all having higher *E′* throughout the complete testing temperature range. This is related to the nanofillers’ nucleating properties on the polymers and it enhancing their stiffness (Figure 11b), as can be seen, for example, comparing PLLA/PVDF (80/20) with the sample with 0.5 wt.% INT-WS_2_, where the room temperature modulus increased 27% from 2274 MPa.

From Figure 12a of the *tan δ* curves, it can be seen that the immiscible blends all presented individual *T*_g_ transitions at temperatures characteristic of the pure PVDF and PLLA polymers, though in the case of PLLA, the *T*_g_ peak decreased slightly and broadened, possibly related to its partial miscibility with PVDF at the interface between the two polymers or its nuclei at this location also increasing chain mobility. The mobility of the PLLA chain segments were improved with INT-WS_2_ addition as can be seen with an increase in the *tan δ* peak of 60/40-INT (Figure 12b) compared to the same blend without nanofiller. The position (glass transition temperature) and height of the *tan δ* peak are associated with segmental mobility. The decrease in *T*_g_ means the enhancement of chain segment mobility and the increase in the height of the *tan δ* peak reveals the rise in segmental mobility. Both the decrease in *T*_g_ and the increase in the height of *tan δ* indicate that the blend nanocomposites had high mobility of chain segments.

## 4. Conclusions

This investigation provides evidence of the successful preparation and performance of new multifunctional biopolymer blend nanocomposites containing poly(l-lactic acid) (PLLA), poly(vinylidene fluoride)(PVDF), and 1D-TMDCs WS_2_. The addition of PVDF and inorganic nanotubes was found to be efficient as an alternative route to produce advanced PLLA/PVDF blend nanocomposites processed via the widely used melt processing. The dispersion of the nanofiller from SEM was observed to be dispersed well and helped modify the blend interface morphology. The PVDF temperature of crystallization was higher and its rate faster than PLLA despite them having comparable melting points. Its effect on PLLA thermal stability was minimal, though it did increase the rate of PLLA crystallization. The incorporated nanofiller INT-WS_2_ had a nucleating effect on both PLLA and PDVF, though it was more prominent on PLLA and PLLA-rich blends. This effect also led to higher enthalpies of crystallization and cold-crystallization omission, but with the final crystalline structure maintained. Finally, the nanocomposite blends with a high PLLA content demonstrated significant mechanical improvements compared to blends without filler, with the possible outlook of these materials being used as medical implants. The use of PLLA and PVDF ensured biocompatibility of the composite, and the presence of inorganic nanotubes additionally provided it with mechanical strength and processability.

## Figures and Tables

**Table 1:** TGA parameters of different PLLA/PVDF/INT-WS_2_ blend nanocomposites based on PLLA, PVDF, and INT-WS_2_.

Material	*T*_i_ (°C)	*T*_10_ (°C)	*T*_max_ (°C)	*R*_max_ (%/°C)
PLLA	325.6	347.0	381.0	29.01
80/20	327.4	351.9	378.2/480.7	23.85/05.34
60/40	332.8	353.7	376.4/488.0	19.00/12.21
40/60	338.3	360.0	377.3/488.0	12.75/22.97
20/80	346.5	368.2	375.5/481.6	8.02/23.73
PVDF	420.9	453.5	478.4	40.34
PLLA-INT	325.8	349.0	377.4	29.39
80/20-INT	327.4	351.9	378.0/489.2	22.61/05.58
60/40-INT	330.2	352.0	377.1/485.3	18.39/09.34
40/60-INT	336.4	358.3	374.8/482.0	13.27/14.96
20/80-INT	344.8	367.1	371.9/476.5	6.95/23.86
PVDF-INT	396.6	437.0	454.5	17.68

*T*_i_: temperature for 2% weight loss; *T*_10_: temperature for 10% weight loss; *T*_max_: temperature corresponding to the maximum rate of weight loss; and *R*_max_: rate of maximum decomposition.

**Table 2:** DSC parameters of different PLLA/PVDF/INT-WS_2_ blend nanocomposites based on PLLA, PVDF, and INT-WS_2_.

Material	*T*_c, PLLA_ (°C)	∆*H*_c, PLLA_ (J/g)	*T*_c, PVDF_ (°C)	∆*H*_c, PVDF_ (J/g)	*T*_cc, PLLA_ (°C)	∆*H*_cc_ (J/g)	*T*_m_ (°C)	∆*H*_m_ (J/g)
PLLA	92.2	8.0	-	-	104.2	27.9	166.1	47.4
80/20	111.3	43.4	133.8	-	-	-	161.0/166.9	53.0
60/40	114.1	41.6	130.0	-	-	-	164.5/139.9	56.0
40/60	113.3	13.0	134.5	25.1	-	-	169.0	45.4
20/80	114.8	8.0	134.0	36.4	-	-	166.0/168.7	46.8
PVDF	-	-	133.1	48.4	-	-	166.2/169.3	48.9
PLLA-INT	117.6	49.1	-	-	-	-	163.9/168.3	52.2
80/20-INT	112.5	54.6	130.6	-	-	-	164.0/169.4	49.2
60/40-INT	114.5	23.9	134.3	4.3	-	-	164.2/169.2	54.4
40/60-INT	109.6	16.7	134.1	29.0	-	-	166.4/170.2	44.4
20/80-INT	115.0	5.8	133.3	37.9	-	-	166.4/169.0	48.4
PVDF-INT	-	-	134.6	48.0	-	-	169.8	47.2

*T*_c_: crystallization temperature; ∆*H*_c_: crystallization entalphy; *T*_cc_: cold-crystallization temperature; ∆*H*_cc_: cold-crystallization entalphy; *T*_m_: melting temperature; ∆*H*_m_: melting entalphy.

**Table 3:** DMA parameters of different PLLA/PVDF/INT-WS_2_ blend nanocomposites based on PLLA, PVDF, and INT-WS_2_.

Material	*E′*_25°C_ (GPa)	*T*_g__, PVDF_ (°C)	*T*_g, PLLA_ (°C)
PLLA	3127	-	55
80/20	2274	-	54
60/40	2055	−36	57
40/60	1985	−37	54
20/80	1776	−38	50
PVDF	1560	−37	-
PLLA-INT	3640	-	54
80/20-INT	2886	−37	53
60/40-INT	2415	−35	53
40/60-INT	2293	−39	56
20/80-INT	2154	−37	-
PVDF-INT	1923	−37	-

## Data Availability

The data presented in this study are available on request from the corresponding author.
